# Insulinase-like Protease 1 Contributes to Macrogamont Formation in Cryptosporidium parvum

**DOI:** 10.1128/mBio.03405-20

**Published:** 2021-03-09

**Authors:** Rui Xu, Yaoyu Feng, Lihua Xiao, L. David Sibley

**Affiliations:** a Department of Molecular Microbiology, Washington University School of Medicine, St. Louis, Missouri, USA; b School of Resources and Environmental Engineering, East China University of Science and Technology, Shanghai, China; c Center for Emerging and Zoonotic Diseases, College of Veterinary Medicine, South China Agriculture University, Guangzhou, China; Albert Einstein College of Medicine

**Keywords:** CRISPR/Cas9, active-site mutant, cryptosporidiosis, oocyst shedding, pathogenesis, sexual development

## Abstract

The apicomplexan parasite Cryptosporidium parvum contains an expanded family of 22 insulinase-like proteases (INS), a feature that contrasts with their otherwise streamlined genome. Here, we examined the function of INS1, which is most similar to the human insulinase protease that cleaves a variety of small peptide substrates. INS1 is an M16A clan member and contains a signal peptide, an N-terminal domain with the HXXEH active site, followed by three inactive domains. Unlike previously studied C. parvum INS proteins that are expressed in sporozoites and during merogony, INS1 was expressed exclusively in macrogamonts, where it was localized in small cytoplasmic vesicles. Although INS1 did not colocalize with the oocyst wall protein recognized by the antibody OW50, immune-electron microscopy indicated that INS1 resides in small vesicles in the secretory system. Notably, these small INS1-positive vesicles were often in close proximity to large OW50-positive vacuoles resembling wall-forming bodies, which contain precursors for oocyst wall formation. Genetic deletion of INS1, or replacement with an active-site mutant, resulted in lower formation of macrogamonts *in vitro* and reduced oocyst shedding *in vivo*. Our findings reveal that INS1 functions in the formation or maturation of macrogamonts and that its loss results in attenuated virulence in immunocompromised mice.

## Abstract (precis)

Cryptosporidiosis is a debilitating diarrheal disease in young children in developing countries. The absence of effective treatments or vaccines makes this infection very difficult to manage in susceptible populations.

## INTRODUCTION

*Cryptosporidium* spp. are apicomplexan parasites that cause diarrheal disease in humans and animals. Human infection is primarily caused by two species, Cryptosporidium parvum, which also infects agricultural ruminant animals and is zoonotic, and C. hominis, which is spread human to human (1). Cryptosporidiosis was recognized as one of the top three causes of severe diarrhea in children younger than 2 years of age in developing countries, as reported by the Global Enteric Multi-Center Study (2). Infection in early life is also associated with lasting defects in development even after infections subside (3). Nitazoxanide is the only FDA-approved drug for the treatment of cryptosporidiosis. However, it has a limited effect in immunocompromised individuals and is not approved for use in children under the age of two (4). There are currently no effective vaccines for C. parvum, and fundamental studies on parasite biology and host-pathogen interactions are needed to identify potential therapeutic and vaccine targets.

The entire life cycle of *Cryptosporidium* occurs in a single host, leading to efficient fecal-oral transmission (5). Following the ingestion of oocysts, sporozoites emerge and invade intestinal epithelial cells, where they develop in a unique vacuole formed at the apex of the host cell (6). The parasite initially grows as a trophozoite before undergoing multiple rounds of asexual replication during merogony (7, 8). The parasite then differentiates to the sexual phase and develops as macrogamonts or microgamonts that begin appearing after 44 to 48 h postinfection (hpi) (7, 8). However, in transformed cell lines grown *in vitro*, the infection does not progress and parasite numbers gradually decline (9). Comparison of *in vitro* cultures in adenocarcinoma cell lines to the developmental process that occurs in the intestine of mice revealed that the block to complete development *in vitro* is due to a lack of fertilization despite the fact that both gametocyte forms develop normally (7).

Biological investigations of *Cryptosporidium* have been hampered by limitations in experimental platforms for *in vitro* growth. Despite this limitation, *in vitro* propagation systems have been used to generate antibodies that identify different stages of C. parvum (10), leading to a better understanding the life cycle (8). Recent developments have also provided systems that allow complete development of infectious oocysts in stem cell-derived cultures *in vitro* (11, 12). Finally, advances in CRISPR/Cas9 technology have allowed genetic modification in C. parvum to tag genes for localization and disrupt them to study function (13). Despite these advances, we lack an understanding of the function of most genes in *Cryptosporidium*, many of which have no orthologues outside the genus or are specific for the phylum Apicomplexa (14, 15).

The genome of C. parvum is highly streamlined, with short intergenic regions, limited introns, and the loss of many metabolic pathways (16). Greater than 98% of genes in C. parvum are present as a single copy, and only a limited number of multigene families include insulinase-like proteases (INS), predicted secretory proteins containing the amino acid sequence MEDLE, and mucin-type glycoproteins (17). INS proteins belong to the M16 family of metallopeptidases that play diverse roles in cells, and they can be found in the cytosol, organelles, and even the cell surface (18). M16 metalloproteases typically bind zinc as part of their active site (HXXEH), and they cleave short polypeptides, the size of which is constrained by a conserved small barrel fold that forms the catalytic chamber (19). The apicomplexan parasite Toxoplasma gondii contains ∼50 metalloproteases, including 11 members of the M16 clan (20), several of which are found in secretory organelles implicated in host cell interactions (21, 22). In Plasmodium falciparum, the M16 metalloprotease falcilysin participates in both hemoglobin degradation and the processing of transit peptides for apicoplast proteins (23, 24). Similarly, other M16 family members are known for their roles in processing transit peptides for mitochondria and chloroplasts (18).

The C. parvum genome contains 22 members of the M16 family of metallopeptidases. Ten of these contain the active site HXXEH, indicating they act as enzymes in the parasite, although none of their substrates have been defined. Previous studies have shown that antibodies against INS20-19 (although originally annotated as two genes, later assemblies collapsed these into a single gene), INS15, or INS5 inhibit parasite invasion of host cells *in vitro*, suggesting they are involved in processing substrates important for host cell recognition or entry (25–27). In the present study, we focused on INS1, which is present among all *Cryptosporidium* spp. INS1 is a classic M16A family member with one active functional motif, HXXEH, followed by three inactive domains. We demonstrate that INS1 is expressed exclusively in macrogamonts of C. parvum, where it localizes to small transport vesicles. Deletion of INS1, or replacement with an inactive-site mutant, reduced formation of macrogamonts *in vitro* and reduced oocyst shedding and decreased virulence in immunocompromised mice.

## RESULTS

### Epitope tagging of C. parvum INS1.

**FIG 1:**
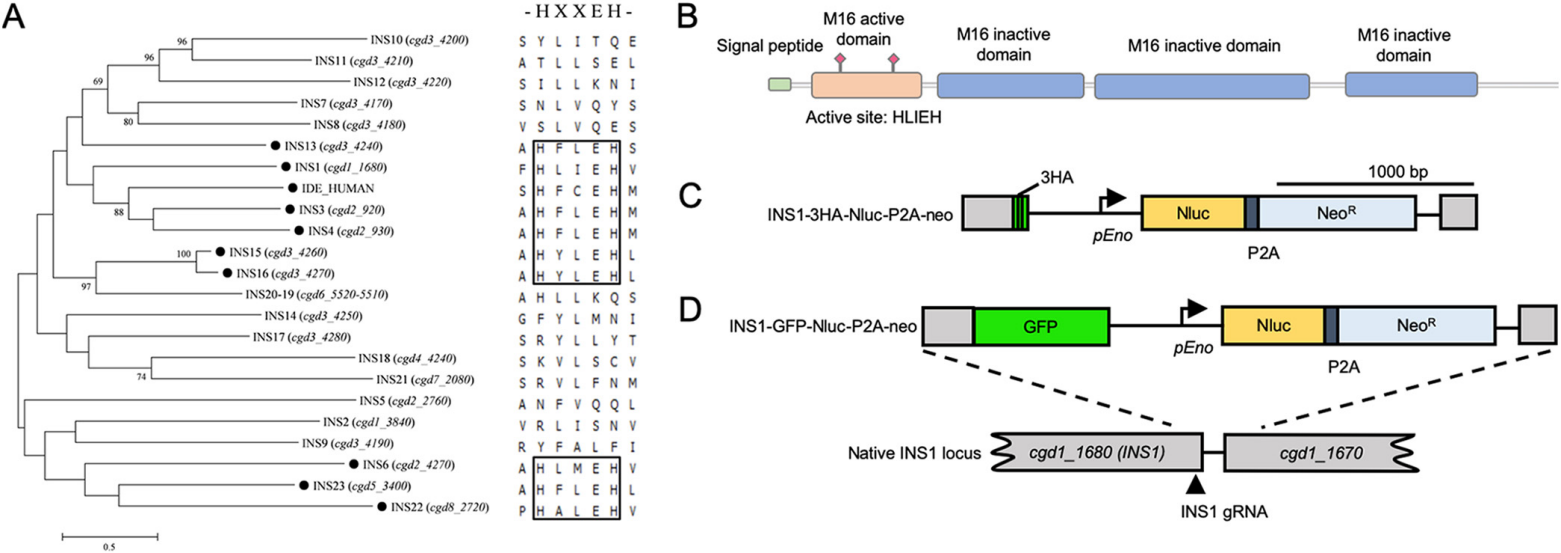
Insulinase-like proteases in Cryptosporidium parvum and construction of C. parvum INS1-3HA and INS1-GFP strains. (A) Phylogenetic relationship of C. parvum insulinase-like protease family and human insulinase. The tree was constructed by a maximum likelihood analysis with 1,000 replications for bootstrapping. The active site HXXEH in each INS was defined by multiple alignments. The black dot indicates proteins with a predicted active site. Scale, 50 changes per 100 residues. (B) Domain architecture of INS1 showing the presence of one M16 active domain containing the active site HLIEH and three inactive domains. (C) Diagram of INS1-3HA tagging strategy. Construct was designed to add a 3HA tag and Nluc-P2A-Neo^R^ cassette at the C terminus of INS1 (*cgd1_1680*). P2A, split peptide. INS1 gRNA marks the site of guide RNA homology. (D) Diagram of INS1-GFP tagging strategy. Construct was designed to add a GFP tag and Nluc-P2A-Neo^R^ cassette at the C terminus of INS1 (*cgd1_1680*). P2A, split peptide; INS1 gRNA, site of guide RNA homology.

We compared the sequences of the 22 members of the M16 metalloprotease family present in C. parvum using a neighbor-joining phylogenetic analysis. For comparison, we also included the human insulinase gene IDE (P14735). Phylogenetic analysis indicated that INS1, encoded by the *cgd1_1680* gene, is most similar to IDE (Fig. 1A). INS1 contains a signal peptide and has four domains, one active domain containing the zinc-binding motif HLIEH and three inactive domains, consistent with INS1 belonging to the M16A clan (Fig. 1B). To investigate the cellular localization of INS1, we used CRISPR/Cas9 genome editing to tag INS1 with a triple hemagglutinin (3HA) epitope tag at the C terminus. The tagging construct also contained a selection cassette consisting of nanoluciferase (Nluc) cotranscribed with neomycin resistance (Neo^r^) driven by an enolase promoter (13) (Fig. 1C). Similar to methods described previously, the Nluc and Neo proteins were separated by a split peptide motif (P2A) (12). This tagging construct was cotransfected with a CRISPR/Cas9 plasmid containing a single guide RNA sequence (sgRNA) located in the C terminus of the gene. To create parasites with INS1 fused to green fluorescent protein (GFP), we replaced the 3HA tag with GFP and used the same INS1 sgRNA plasmid (Fig. 1D).

### Generation of stable transgenic INS1 parasites.

**FIG 2:**
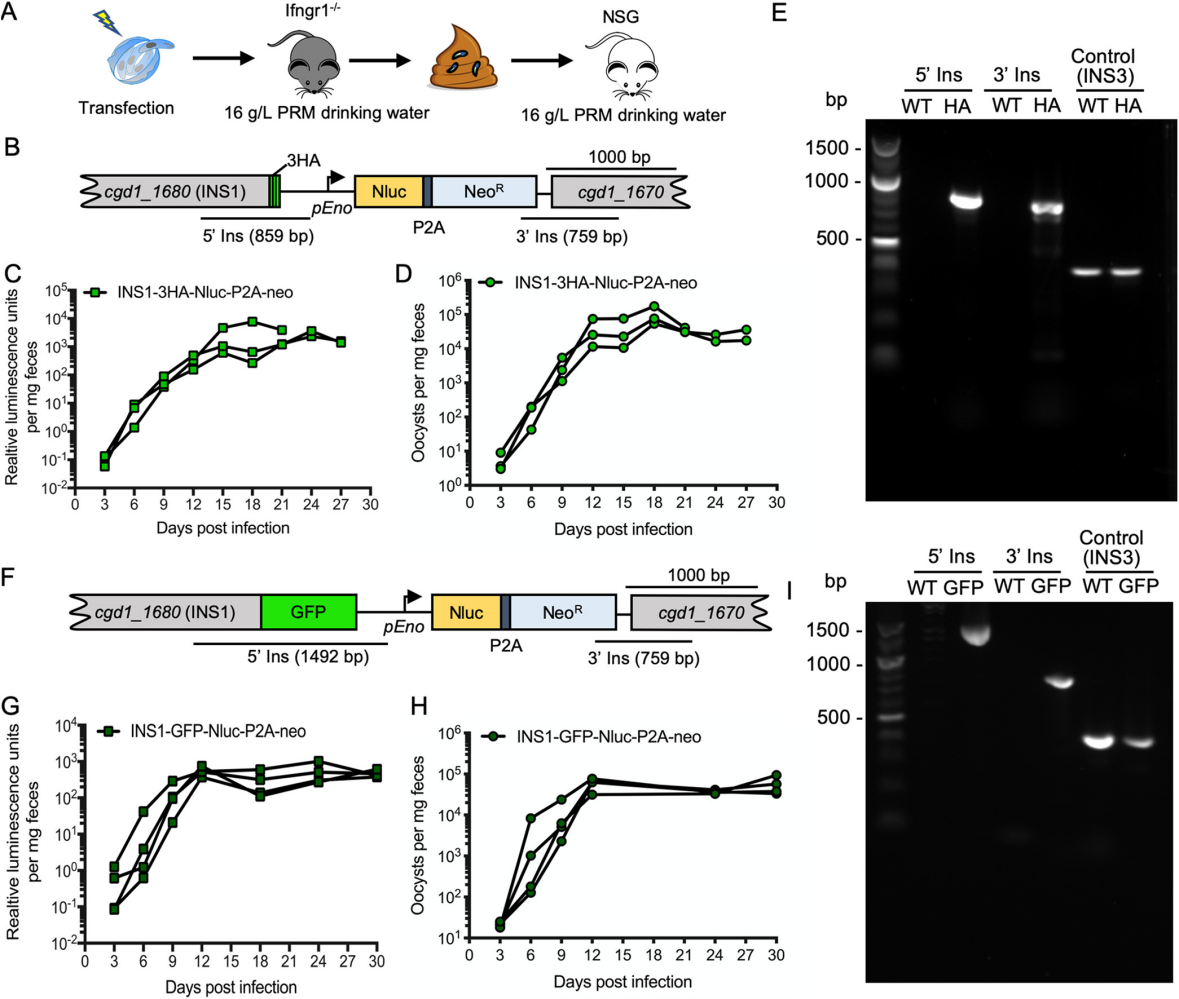
Amplification of transgenic parasites in immunocompromised mice. (A) Amplification strategy for obtaining tagged INS1 parasites. Approximately 5 × 10^7^ sporozoites were cotransfected with 50 μg tagging plasmid and 30 μg CRISPR/Cas9 plasmid per cuvette. Each Ifngr1^−/−^ mouse was gavaged with 200 μl 8% sodium bicarbonate solution 5 min before being infected by gavaged with 2.5 × 10^7^ transfected sporozoites in 100 μl of DPBS. A second round of selection was conducted in NSG mice. Each mouse was gavaged with fecal slurry containing 2 × 10^4^ oocysts obtained at 13 dpi from the first round of selection. All mice received 16 g/liter paromomycin drinking water from the first day postinfection (dpi) for the duration of the experiment. (B) Diagram of the INS1-3HA-tagged locus in stable transgenic parasites. C. parvum was cotransfected with INS1-3HA-Nluc-P2A-neo tagging plasmid and CRISPR/Cas9 plasmid containing an INS1 sgRNA specific to the *INS1* locus. (C) Relative luminescence per milligram of feces from transgenic C. parvum oocysts. Each data point represents a single pellet, and each connecting line represents an individual infected NSG mouse from round two amplification of transfected parasites. (D) The number of oocysts per milligram of feces was measured by qPCR. Each data point represents a single pellet, and each connecting line represents an individual NSG mouse from round two of amplification of transfected parasites. (E) PCR analysis of INS1-3HA oocysts amplified in mice from round two. WT, wild type. HA, INS1-3HA transgenic parasites. The product 5′ Ins is specific for the 5′ CRISPR targeting site of INS1-3HA. The product 3′ Ins is specific for the 3′ CRISPR targeting site of INS1-3HA. Control, product is specific to the INS3 locus. Primers are defined in Table S1. (F) Diagram of the INS1-GFP-tagged locus in stable transgenic parasites. C. parvum was cotransfected with the INS1-GFP-Nluc-P2A-neo tagging plasmid and CRISPR/Cas9 plasmid containing an INS1 gRNA specific to the *INS1* locus. (G) Relative luminescence per milligram of feces from transgenic C. parvum oocysts. Each data point represents a single pellet, and each connecting line represents an individual NSG mouse infected with INS1-GFP parasites from round two. (H) The number of oocysts per milligram of feces was measured by qPCR. Each data point represents a single pellet, and each connecting line represents an individual NSG mouse infected with INS1-GFP parasites from round two. (I) PCR analysis of INS1-GFP oocysts amplified in mice from round two. WT, wild type. GFP, INS1-GFP transgenic parasites. The product 5′ Ins is specific for the 5′ CRISPR targeting site of INS1-GFP. The product 3′ Ins is specific for the 3′ CRISPR targeting site of INS1-GFP. Control, product is specific to the *INS3* locus. Primers are defined in Table S1.

Excysted sporozoites were electroporated with INS1-3HA-Nluc-P2A-neo tagging plasmid and CRISPR/Cas9 plasmid containing the INS1 sgRNA. Following electroporation, sporozoites were used to infect Ifngr1^−/−^ mice. Oocyst shedding reached a peak level 13 days postinfection (dpi), and all of the mice succumbed to infection by day 15, indicating the tagged line was not attenuated (see Fig. S1 in the supplemental material). Feces were collected from the first round of mice, and a slurry was gavaged into a group of *Nod scid gamma* (NSG) mice to obtain the larger numbers of transgenic oocysts for purification. All mice were treated with 16 g/liter paromomycin in drinking water for selection of stable transgenic parasites (Fig. 2A), as described previously (13). The signal of luminescence from the nLuc gene increased in fecal pellets from 6 dpi (days postinfection), with a peak value on 12 dpi (Fig. 2C). Quantification of oocysts in feces by qPCR paralleled the increase in luciferase activity (Fig. 2D). PCR analysis of oocysts collected from the mice confirmed that the INS1-3HA tagging cassette had correctly inserted into the *INS1* locus, as shown using diagnostic primers that only amplify from the correct transgenic arrangement (Fig. 2B and E). To obtain a tagged INS1-GFP strain, we used the same strategy to amplify the INS1-GFP transgenic parasites. In the first round of infection in Ifngr1^−/−^ mice, all of the animals succumbed by day 15, indicating the tagged line was not attenuated (Fig. S1). In the second round of amplification in NSG mice, luminescence and oocyst numbers measured from infected mouse feces increased on 6 dpi and remained elevated 1 month (Fig. 2G and H). PCR analysis confirmed that the INS1-GFP strain had correctly inserted into the *INS1* locus (Fig. 2F and I). Fecal pellets were collected every day from 12 to 30 dpi for purification of transgenic parasites.

10.1128/mBio.03405-20.2 FIG S1 Selection of transgenic parasites in Ifngr1^−/−^ mice. (A) Detection of NanoLuc expression from transgenic C. parvum oocysts in mouse fecal pellets. Each Ifngr1^−/−^ mouse was gavaged with 2.5 × 10^7^ transfected sporozoites of the indicated lines, and mice received 16 g/liter paromomycin drinking water from the first day postinfection (dpi) for the duration of the experiment. For the INS1-3HA and INS1-GFP groups, the data plotted here are averages and variances from two animals. For the Δ*ins1* and *INS1^m^* groups, the data shown are the averages and SD. *, animals that died. (B) The number of oocysts per milligram of feces was measured by qPCR. Animals in each group are the same as those shown in panel A. For the INS1-3HA and INS1-GFP groups, the data plotted here are the averages and variances from two animals. For the Δ*ins1* and *INS1^m^* groups, the data shown are the averages and SD. *, animals that died. (C) Survival curve of Ifngr1^−/−^ mice infected with different transgenic parasites. Animals correspond to those shown in panels A and B. For the WT group, four Ifngr1^−/−^ mice were gavaged with 2 × 10^4^ oocysts. Download FIG S1, DOCX file, 0.4 MB. Copyright © 2021 Xu et al. 2021 Xu et al. https://creativecommons.org/licenses/by/4.0/ This content is distributed under the terms of the Creative Commons Attribution 4.0 International license.

10.1128/mBio.03405-20.1 TABLE S1 Oligonucleotides used in this study. Download Table S1, XLSX file, 0.01 MB. Copyright © 2021 Xu et al. 2021 Xu et al. https://creativecommons.org/licenses/by/4.0/ This content is distributed under the terms of the Creative Commons Attribution 4.0 International license.

### INS1 is expressed in macrogamonts.

**FIG 3:**
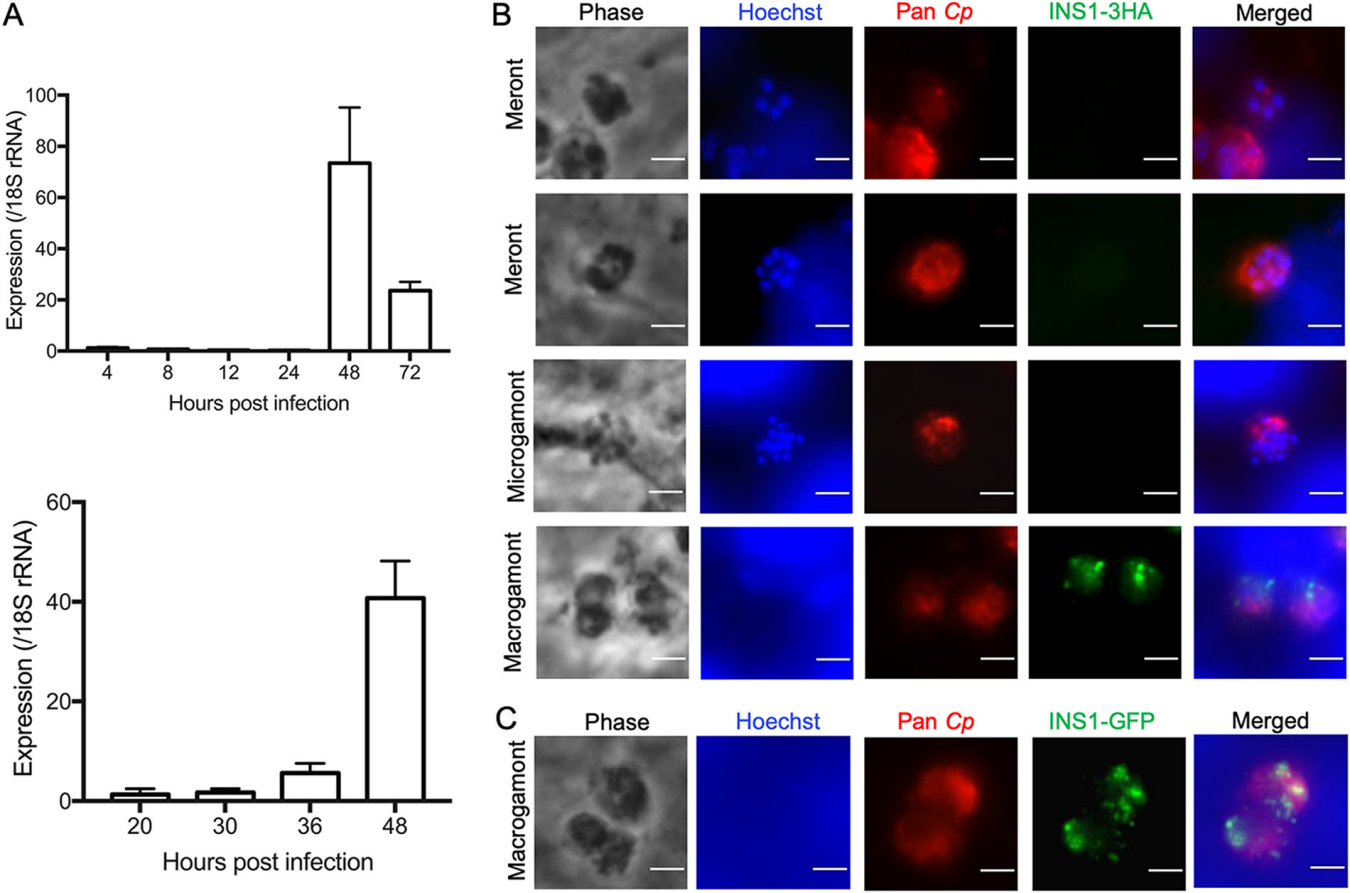
Transcription and expression of C. parvum INS1 *in vitro*. (A) Relative transcription level of the INS1 gene (*cgd1_1680*) at specified times postinfection, as determined by reverse transcription-quantitative PCR. HCT-8 cells were infected with C. parvum oocysts and cultured for specific time points, and RNA was collected from three wells per time point. Gene expression profiles are from two separate experiments with different time points. Data from the *Cryptosporidium* 18S rRNA gene were used in data normalization. Values are plotted as the means ± standard deviations (SD). (B) Immunofluorescence staining of transgenic INS1-3HA parasites. HCT-8 cells were infected with INS1-3HA oocysts. After 48 hpi, coverslips were fixed and stained with rat anti-HA followed by goat anti-rat IgG Alexa Fluor 488, rabbit pan-Cp followed by goat anti-rabbit IgG Alexa Fluor 568, and Hoechst for nuclear staining. Scale bars, 2 μm. (C) Immunofluorescence staining of INS1-GFP parasites. HCT-8 cells were infected with INS1-GFP oocysts. After 48 hpi, coverslips were fixed and stained with rabbit anti-GFP followed by goat anti-rabbit IgG Alexa Fluor 488, rat pan-Cp followed by goat anti-rat IgG Alexa Fluor 568, and Hoechst for nuclear staining. Scale bars, 2 μm.

To examine the expression of *INS1* during intracellular development of C. parvum *in vitro*, we infected HCT-8 cells with wild-type oocysts and tested expression by reverse transcription-quantitative PCR (RT-qPCR) at different time points. We used two slightly different sets of RNA samples designed to cover the time range of the development of asexual and sexual stages, as described previously (8). The *INS1* gene showed no transcription before 30 h postinfection (hpi) but was upregulated at 36 hpi and reached the highest level at 48 hpi before declining at 72 hpi (Fig. 3A). The sexual stages of C. parvum first appear ∼36 hpi (7, 8), and the positive signal for *INS1* at this time point indicates that *INS1* was expressed in either macrogamonts or microgamonts. To visualize the stage-specific expression and localization of INS1, we performed immunofluorescence assays (IFA) using INS1-3HA and INS1-GFP transgenic parasites. Neither the anti-HA antibody nor anti-GFP antibody detected any staining above background in asexual stages of C. parvum, consistent with the very low level of transcription of INS1 during these stages. Instead, INS1 was only detected in the macrogamont stage, where it appeared as punctate staining, while microgamonts remained negative (Fig. 3B and C).

**FIG 4:**
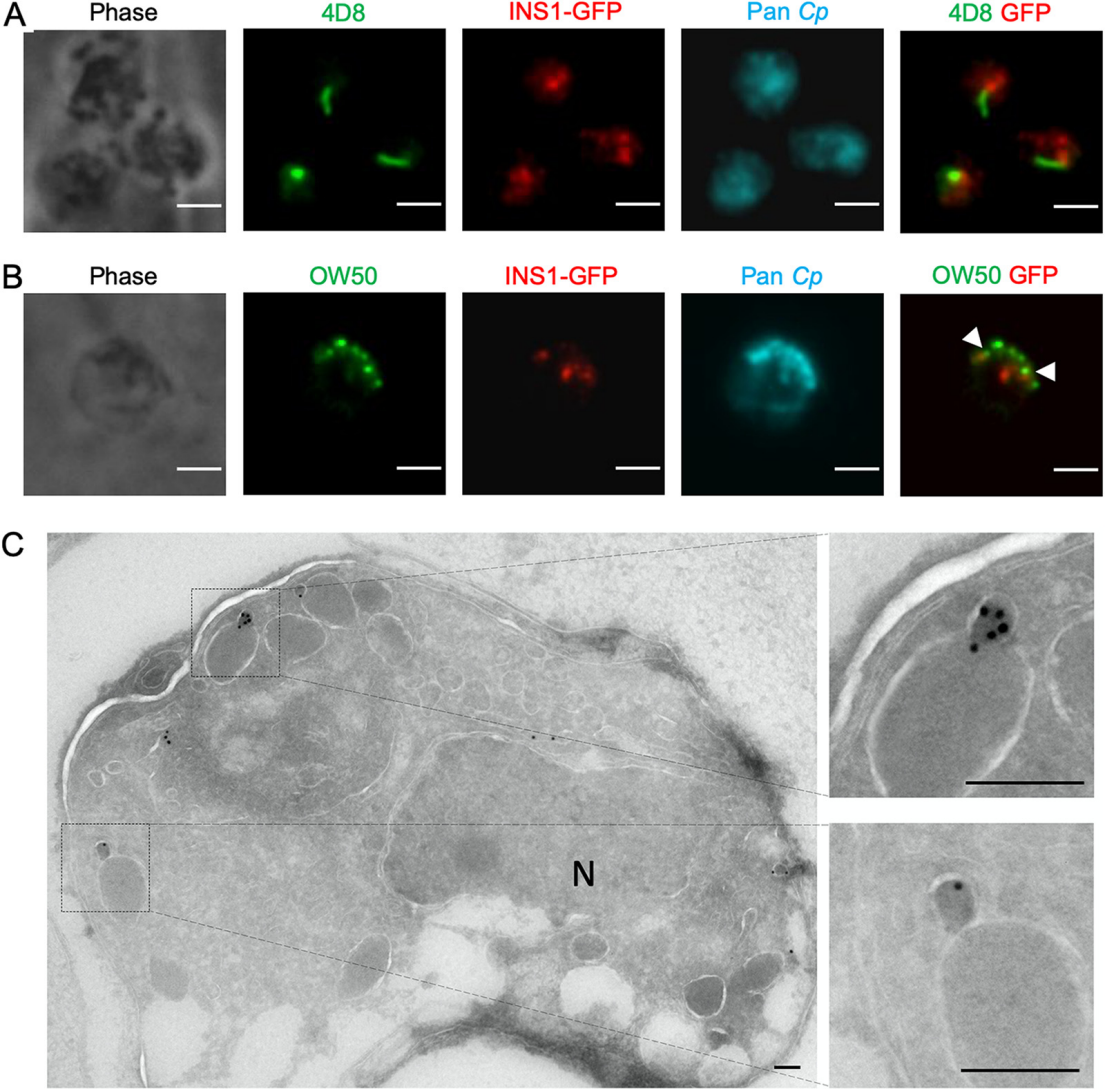
Expression of INS1 in different life cycle stages of C. parvum. (A) Immunofluorescence staining of macrogamont-specific MAb 4D8 in INS1-GFP parasites. HCT-8 cells were infected with INS1-GFP oocysts. After 48 hpi, coverslips were fixed and stained with mouse MAb 4D8 followed by goat anti-mouse IgM Alexa Fluor 488, rabbit anti-GFP followed by goat anti-rabbit IgG Alexa Fluor 568, rat pan-Cp followed by goat anti-rat IgG Alexa Fluor 647, and Hoechst for nuclear staining. Scale bars, 2 μm. (B) Immunofluorescence staining of OW50 in INS1-GFP parasites. Arrowheads indicate staining of OW50-positive vesicles that were in close proximity to INS1. HCT-8 cells were infected with INS1-GFP oocysts. After 48 hpi, coverslips were fixed and stained with mouse MAb OW50 followed by goat anti-mouse IgG Alexa Fluor 488, rabbit anti-GFP followed by goat anti-rabbit IgG Alexa Fluor 568, rat pan-Cp followed by goat anti-rat IgG Alexa Fluor 647, and Hoechst for nuclear staining. Scale bars, 2 μm. (C) Transmission electron micrographs of macrogamont of INS1-GFP parasites. HCT-8 cells were infected with INS1-GFP oocysts. After 48 hpi, cells were fixed and stained with rabbit anti-GFP followed by 18-nm colloidal gold goat anti-rabbit IgG. Two images on the right are enlarged sections of the image on the left, as indicated by the dotted lines. N, nucleus. Scale bars, 200 nm.

Only a limited number of stage-specific proteins have been characterized in C. parvum, therefore we investigated two antibodies that have been previously shown to detect proteins that are expressed in macrogamonts. The punctate structures stained positively for INS1 did not colocalize with monoclonal antibody (MAb) 4D8, which recognizes a prominent filament structure of macrogamonts (10) (Fig. 4A). We also examined costaining with MAb OW50, which stains cytoplasmic inclusions called wall-forming bodies that are released to form the oocyst wall (28). INS1 was in clusters distinct from those of OW50; however, these were often in close proximity to each other (Fig. 4B). To explore the ultrastructural localization of INS1, INS1-GFP transgenic parasites were examined by immunoelectron microscopy. INS1 was located in small vesicles within macrogamonts, and these positive compartments were often in proximity to large electron-dense vesicles (Fig. 4C). Based on its timing of expression and localization, these findings suggest that INS1 plays some role in the development of macrogamonts.

### Decreased oocyst shedding in Δ*ins1* parasites.

**FIG 5:**
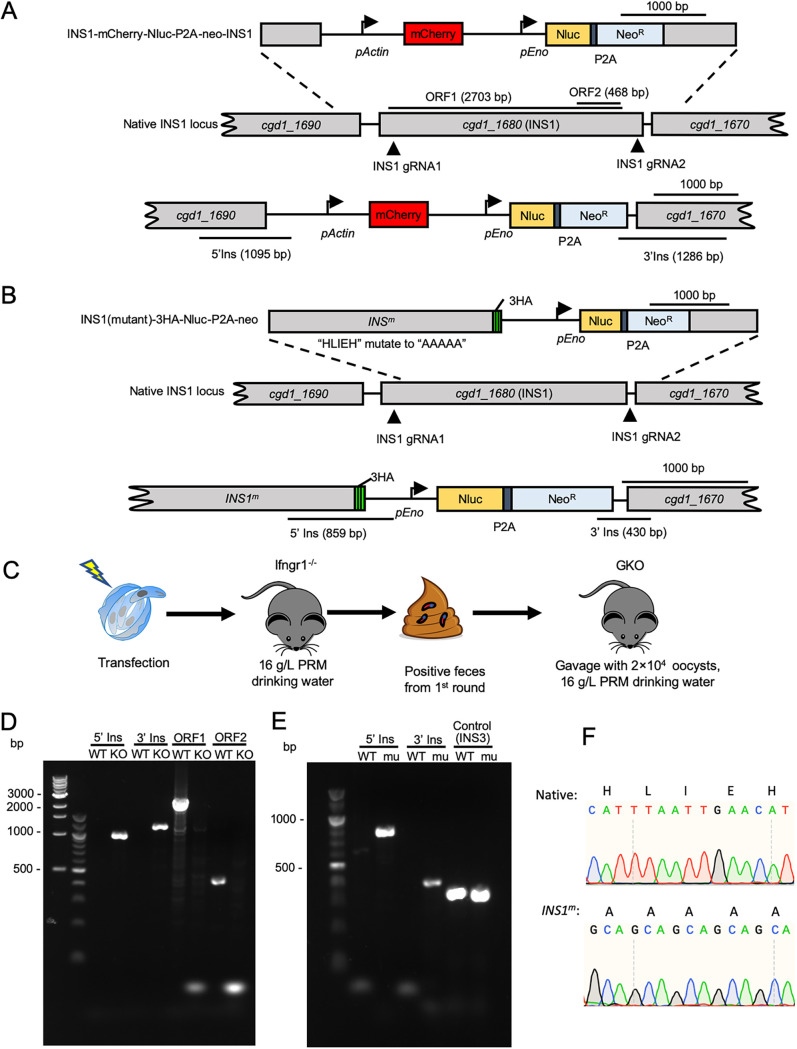
Selection of Δ*ins1* parasites and *INS1^m^* transgenic parasites in immunocompromised mice. (A) Diagram of the strategy to construct Δ*ins1* transgenic parasites. Construct was designed to replace the *INS1* locus with an mCherry and Nluc-P2A-Neo^R^ cassette. The top line shows the targeting construct, the middle line the genomic locus, and the bottom line the successfully targeted transgenic locus. P2A, split peptide; INS1 gRNA, site of guide RNA homology. (B) Diagram of the strategy to construct *INS1^m^* active-site mutants. Construct was designed to make an INS1 point mutation in which the active site HLIEH was mutated to AAAAA and added a 3HA tag and Nluc-P2A-Neo^R^ cassette at the C terminus of INS1 (*cgd1_1680*). The top line shows the targeting construct, the middle line the genomic locus, and the bottom line the successfully targeted transgenic locus. P2A, split peptide; INS1 gRNA, site of guide RNA homology. (C) Selection strategy for obtaining Δ*ins1* or *INS1^m^* transgenic parasites. Transfected sporozoites were gavaged into Ifngr1^−/−^ mice treated with 16 g/liter paromomycin in drinking water. A second round of selection was conducted in GKO mice. Each mouse in round two was gavaged with a fecal slurry containing 2 × 10^4^ oocysts collected at 18 dpi of the first round of selection. (D) PCR analysis of Δ*ins1* oocysts obtained from the second round of amplification. WT, wild type. KO, Δ*ins1* parasite. The product 5′ Ins is specific for the 5′ CRISPR targeting site of the Δ*ins1* parasite. The product 3′ Ins is specific for the 3′ CRISPR targeting site of Δ*ins1* parasite. The product ORF1 detects a 2,703-bp fragment of the *INS1* open reading frame. The product ORF2 detects a 468-bp fragment of the *INS1* open reading frame. (E) PCR analysis of *INS1^m^* oocysts obtained from the second round of amplification. WT, wild type. mu, *INS1^m^* parasite. The product 5′ Ins is specific for the 5′ CRISPR targeting site of *INS1^m^*. The product 3′ Ins is specific for the 3′ CRISPR targeting site of *INS1^m^*. Control, product is specific to the *INS3* locus. (F) Sequence electropherogram of PCR products from native INS1 (top) and active-site mutant *INS1^m^* (bottom) transgenic parasites. Native, the amino acid and nucleotide sequence of the active site in wild-type INS1 parasite. *INS1^m^*, the amino acid and nucleotide sequence of the active site in *INS1^m^* parasite.

To investigate INS1 function in C. parvum, we generated *INS1* knockout (Δ*ins1*) parasites using CRISPR/Cas9. The *INS1* gene was replaced with an mCherry expression cassette driven by the C. parvum actin promoter in a construct that also contained the Nluc-P2A-neo^R^ selection marker described above (Fig. 5A). To ensure complete removal of the *INS1* gene, we used two sgRNA sequences to degenerate double-stranded DNA breaks that flank the gene (Fig. 5A). Sporozoites were electroporated with the INS1-mCh-Nluc-P2A-neo-INS1 plasmid and a CRISPR/Cas9 plasmid containing the two *INS1* sgRNAs (Fig. 5A). Transfected sporozoites were gavaged into a group of Ifngr1^−/−^ mice treated with 16 g/liter paromomycin in the drinking water. Unlike the tagged lines described above, the knockout line was attenuated, and none of the Ifngr1^−/−^ mice succumbed over a 30-day period (Fig. S1). Moreover, the peak leak level of oocyst shedding by the knockout line was 3.5-fold lower than that of the GFP-tagged line at 9 dpi (Fig. S1). After 13 dpi, the feces containing the Δ*ins1* oocysts were collected and gavaged into a group of immunocompromised mice. For this second-round infection, we were interested in confirming the attenuation of this line, so we used Ifng^−/−^ (GKO) mice, which, like Ifngr1^−/−^ mice, are highly sensitive to C. parvum infection (Fig. 5C). PCR analysis confirmed that oocysts shed by GKO mice were Δ*ins1* parasites based on correct 5′ and 3′ flanking sequences created by the loss of the *INS1* gene and insertion of the mCherry expression cassette in its place (Fig. 5D). Additionally, primers to the open reading frame of *INS1* failed to amplify a product from the knockout line, although they easily detected the expected fragments from wild-type parasites (Fig. 5D).

**FIG 6:**
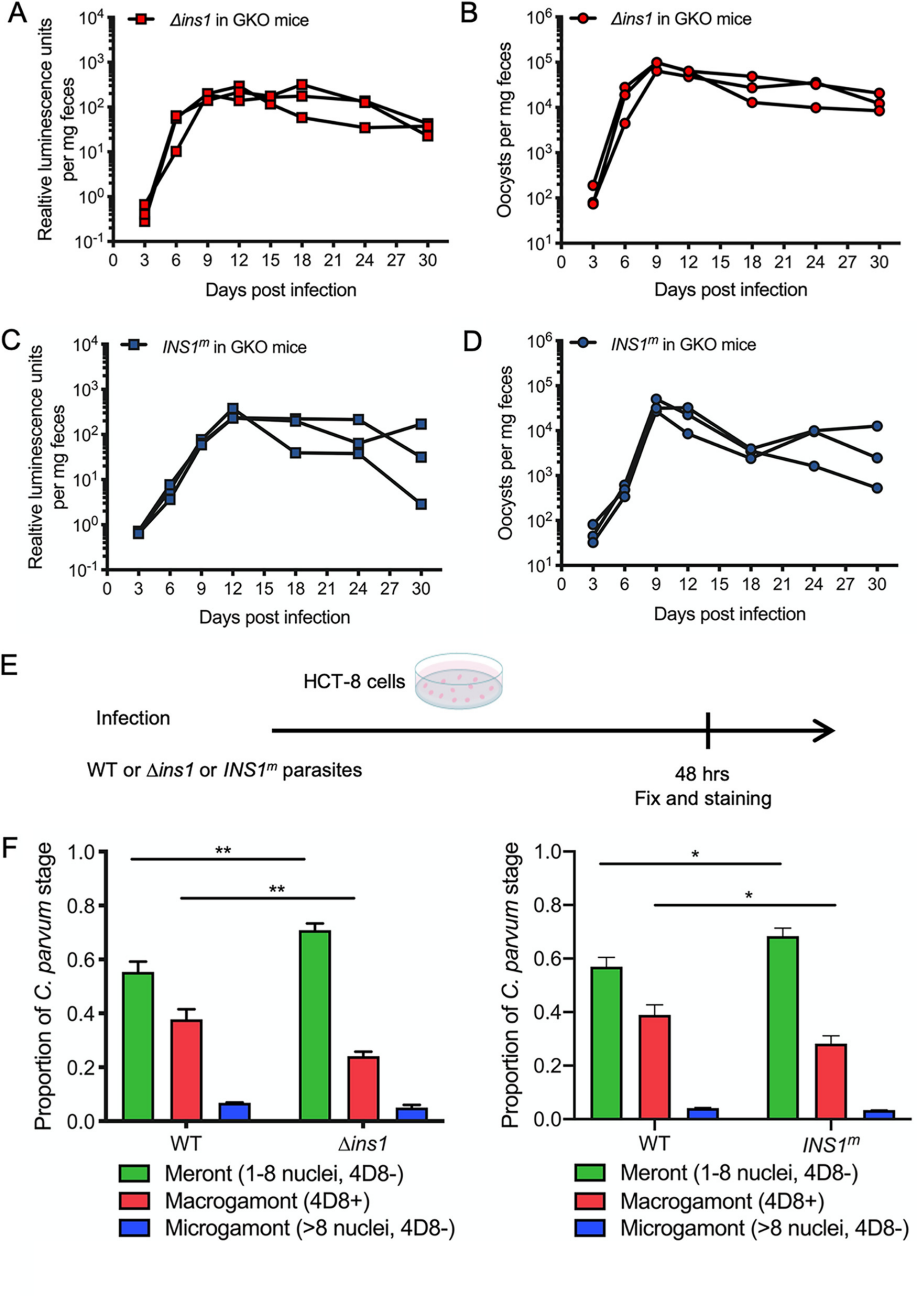
Influence of Δ*ins1* parasites and *INS1^m^* parasites on development of C. parvum *in vivo* and *in vitro*. (A) Relative luminescence of C. parvum per milligram of feces. Each red box represents a single pellet, and each connecting line represents an individual GKO mouse infected with Δ*ins1* parasites from the second round of amplification. (B) The number of oocysts per milligram of feces was measured by qPCR. Each red dot represents a single pellet, and each connecting line represents an individual GKO mouse infected with Δ*ins1* parasites from the second round of amplification. (C) Relative luminescence of C. parvum per milligram of feces. Each blue box represents a single pellet, and each connecting line represents an individual GKO mouse infected with *INS1^m^* parasites from the second round of amplification. (D) The number of oocysts per milligram of feces was measured by qPCR. Each blue dot represents a single pellet, and each connecting line represents an individual GKO mouse infected with *INS1^m^* parasites from the second round of amplification. (E) Outline of the experimental protocol to analyze growth of WT or Δ*ins1* or *INS1^m^* parasites in HCT-8 cells. C. parvum WT or Δ*ins1* or *INS1^m^* parasites were used to infect HCT-8 cells. After 48 hpi, wells were washed, fixed, and labeled with different antibodies. For WT parasites, coverslips were stained with mouse MAb 4D8 that detects macrogamonts followed by goat anti-mouse IgM Alexa Fluor 488, rabbit pan-Cp followed by goat anti-rabbit IgG Alexa Fluor 568, and Hoechst for nuclear staining. For Δ*ins1* parasites, coverslips were stained with mouse 4D8 followed by goat anti-mouse IgM Alexa Fluor 488, rat anti-mCherry followed by goat anti-rat IgG Alexa Fluor 568, rabbit pan-Cp followed by goat anti-rabbit IgG Alexa Fluor 647, and Hoechst for nuclear staining. For *INS1^m^* parasites, coverslips were stained with mouse 4D8 followed by goat anti-mouse IgM Alexa Fluor 488, rabbit pan-Cp followed by goat anti-rabbit IgG Alexa Fluor 568, rat anti-HA followed by goat anti-rat IgG Alexa Fluor 647, and Hoechst for nuclear staining. (F) Quantification of life cycle stages of wild-type, Δ*ins1* (left), or *INS1^m^* (right) parasites. Meronts were identified by their content of 1 to 8 nuclei; macrogamonts were identified by labeling with MAb 4D8; microgamonts were identified by many small nuclei (∼16 nuclei). Each time point represents the average from three biological replicates. The number of parasites was counted from 50 fields of view with a 100× oil objective. Values are plotted as the means ± SD. Statistical analysis was performed using unpaired Student's *t* test for two-sample comparison (*, *P <* 0.05; **, *P <* 0.01).

We then tracked the expansion of the *Δins1* parasites by luciferase measurements and oocyst shedding in the feces in the second round of mice. Both luminescence values and oocyst shedding increased at 6 dpi and reached the peak at 12 dpi (Fig. 6A). Moreover, all mice infected by Δ*ins1* parasites survived the infection and continued to shed oocysts for as long as 1 month, the longest time point we tracked (Fig. 6B).

### INS1 requires active protease activity for function.

We further tested whether INS1 requires its protease activity for function. INS1 contains the active-site HLIEH that is a conserved motif in M16 metalloproteases. We used CRISPR/Cas9 genome editing to alter the INS1 active site from HLIEH to AAAAA while also adding a C-terminal 3HA tag (Fig. 5B). The replacement of the endogenous locus with this active-site mutant template (*INS1^m^*) was guided by two sgRNA sequences located in the N terminus and 3′-untranslated region (UTR) of the gene (Fig. 5B). Sporozoites were electroporated with the INS1(mu)-3HA-Nluc-P2A-neo plasmid, and a CRISPR/Cas9 plasmid containing two *INS1* gRNAs and oocysts were amplified as before. Similar to the Δ*ins1* knockout, this active-site mutant line was attenuated, and none of the Ifngr1^−/−^ mice succumbed over a 30-day period (Fig. S1). Moreover, the peak leak level of oocyst shedding by the mutant line occurred much later, at day 18, and was 26-fold lower than that of the GFP-tagged line that peaked at day 13 (Fig. S1). To confirm this attenuation, we used GKO mice for the second round of infection. PCR analysis demonstrated that oocysts shed by GKO mice contained the correct 5′ and 3′ insertions of the repair template integrated into the *INS1* locus (Fig. 5E). Sanger DNA sequencing of PCR products amplified from *INS1^m^* parasites confirmed the replacement of the wild-type copy with the mutated active site in oocysts recovered from the GKO mice (Fig. 5F). The *INS1^m^* active-site mutants grew with slower kinetics than wild-type parasites, and all infected mice survived infection for as long as 1 month, similar to the Δ*ins1* parasites described above (Fig. 6C and D).

### Inhibition of macrogamont maturation in *Δins1* and *INS1^m^* parasites *in vitro*.

To test the growth abilities of the knockout and active-site mutants *in vitro*, we infected HCT-8 cells with 10^4^ oocysts of each of the mutant versus wild-type parasites and returned them to culture to allow development. After 48 hpi, we fixed cells and labeled them with MAb 4D8 to detect macrogamonts as well as pan-Cp antibody to detect all stages (Fig. 6E). Life cycle stages were then defined on the following bases: meronts were classified by one to eight nuclei but no 4D8 labeling, macrogamonts were classified by a single nucleus and were positive 4D8 labeling, and microgamonts were classified as having 16 nuclei but no 4D8 labeling. Wild-type parasite cultures showed 55% of parasites were meronts, 38% of parasites were macrogamonts, and 7% of parasites were microgamonts at 48 hpi. In contrast, Δ*ins1* cultures showed significantly fewer macrogamonts (24%) with an increase in the proportion of meronts (71%) (Fig. 6F). Similarly, HCT-8 cultures infected with the active-site *INS1^m^* mutant parasites showed significantly fewer macrogamonts (28%) with an increase in the proportion of meronts (68%) (Fig. 6F). Collectively, these findings indicate that INS1 functions to facilitate macrogamont development, and in its absence, oocyst shedding is reduced in immunocompromised mice.

## DISCUSSION

The C. parvum genome contains an expanded family of 22 M16 metalloproteases, the majority of which have unknown functions. Here, we focused on INS1, which is a clan M16A metalloprotease most similar to human insulinase. INS1 contains a signal peptide and an active domain containing the catalytic site HXXEH followed by several inactive domains. Unlike previously described INS proteins that are expressed early in development in C. parvum, INS1 was expressed exclusively in macrogamonts, where it localized to small vesicular structures in the cytosol. Deletion of INS1, or replacement with an active-site mutant, resulted in reduced formation of macrogametocytes *in vitro* and lower oocyst shedding *in vivo*. Our studies reveal that INS1 likely participates in macrogamont formation, and in its absence the parasite forms fewer oocysts *in vivo* and is attenuated in immunocompromised mice.

Metalloproteases are widespread in biology and have been classified into 16 clans that are summarized in the MEROPS database (https://www.ebi.ac.uk/merops/). M16 metalloproteases are characterized by the presence of a zinc-binding motif consisting of the sequence HXXEH (20). M16A and M16C family members contain four domains, only one of which contains the active catalytic site (20). The best known M16 protease is human insulinase, which cleaves a variety of small peptides in different types of cells, consistent with the multiple roles of this enzyme (29). Apicomplexan parasites contain numerous M16 metalloproteases, although their roles have only partially been investigated. Two M16C proteases have been described in Plasmodium falciparum. Falcilysin is involved in hemoglobin degradation and processing of the apicoplast import transit peptides (23, 24), an activity that is also catalyzed by P. falciparum SPP (30). Toxoplasma gondii contains 11 M16 orthologues, and several M16A enzymes found in secretory compartments have been previously studied (20). Toxolysin 1 is found in rhoptries (21), while toxolysin 4 is found in micronemes (22). Both proteases are themselves processed during maturation, but their substrates and the roles these proteases play in their respective secretory compartments are undefined. Toxolysin 4 is refractory to gene disruption (22), while the loss of toxolysin 1 has no effect on growth *in vitro* or virulence *in vivo* (21). Toxolysin 3, encoded by TGME49_257010, is most similar to INS1 by sequence homology. Toxolysin 3 is annotated as a sporozoite development protein (https://toxodb.org/toxo/app), as it is highly expressed in unsporulated and sporulated oocysts (31). In contrast, INS1 is expressed exclusively in macrogamonts, indicating that these M16 metalloproteases have different biological roles.

Of the 22 M16 metalloproteases encoded by the C. parvum genome, 18 of them belong to the M16A family, and several of these have previously been studied, including INS5, INS15, and INS20-19. INS15 and INS20-19 contain signal peptides, suggesting they are in the secretory pathway, while INS5 does not have a predicted signal peptide (25–27). INS20-19 is expressed in sporozoites and localized to an apical compartment (25), while INS15 is expressed in a middle-anterior compartment in sporozoites and merozoites (26). In contrast, INS5 is expressed at lower levels in sporozoites and increases to a peak at 36 to 48 hpi (27). INS5 is present in a punctate pattern in sporozoites and during merogony (27). Antibodies to these proteins have been shown to inhibit parasite invasion *in vitro* (25–27), suggesting they play some role in processing or maturation of substrates involved in host cell recognition. Their similar expression pattern during the life cycle, secretory nature, and ability of antibodies against them to partially neutralize infection suggests that the above-mentioned paralogous INS proteins play partially redundant roles in interacting with the host cell. INS1 is a classic M16A protease containing a signal peptide and an HLIEH active motif in the N-terminal domain followed by three domains that lack this catalytic motif. INS1 has closely related orthologues in other *Cryptosporidium* spp. (32), suggesting it has a conserved function. However, unlike the INS proteins mentioned above, INS1 is not expressed in sporozoites or merozoites and, hence, is unlikely to function in interactions with the host cell during invasion. The phylogenetic analysis of INS genes in C. parvum identified several genes that are closely related to INS1. These include INS3 (encoded by *cgd2_920*), which is also more highly expressed in macrogamonts (7), although it does not contain a signal peptide, suggesting that it has a different role than INS1. Likewise, the related protein INS4 (encoded by *cgd2_930*), which is expressed much earlier at 24 hpi (7), also lacks a signal peptide, suggesting it has a different role than INS1.

INS1 was not detected in sporozoites or during merogony, but expression was strongly upregulated at 36 to 48 hpi, when the sexual stages are formed (8). Consistent with this pattern, previous studies have shown that INS1 and INS3 are highly expressed in macrogamonts *in vivo* and in *in vitro* culture (7). Tagging with the HA epitope or a GFP fusion indicated that INS1 is expressed exclusively in macrogamonts, where it is localized to small vesicular structures in the cytosol. INS1 was not colocalized with 4D8 antibody, which recognizes a unique striated fiber in macrogamonts (10). Additionally, INS1 only partially colocalized with OW50, which recognizes large punctate vesicles in the cytosol of macrogamonts and the wall in mature oocysts (12). When examined by immunoelectron microscopy, INS1 was found in small vesicular structures that were often adjacent to large electron-dense vesicles. These large vesicles resemble wall-forming bodies, which are large cytoplasmic vesicles that contain proteins involved in the formation of the oocyst wall (33). Attempts to label OW50, a component of wall-forming bodies and the oocyst wall, in combination with INS1 by immunoelectron microscopy were not successful. Nonetheless, the proximity of small vesicles containing INS1 to the larger vesicles suggests that INS1 is involved in the processing or trafficking of constituents of the wall-forming body vesicles and that disruption of this process impairs macrogamont maturation and subsequent oocyst formation. Like other insulinase enzymes (19), INS1 is predicted to have a small folded barrel containing the active site that is only capable of accommodating small peptides ranging from 3 to 6 kDa. As such, INS1 is likely to process small peptides, possibly resulting from trimming of secretory proteins by other peptidases in the secretory pathway.

The deletion of INS1, or replacement with the *INS1^m^* active-site mutant, did not affect asexual replication *in vitro* or completely block development, indicating that INS1 is not essential for growth. Instead, the loss of INS1 resulted in the reduced formation of macrogamonts *in vitro* and lower oocyst shedding *in vivo*. In human insulinase, replacement of E^111^ in the HXXEH active site with Q^111^ inactivates catalysis (34, 35). Similarly, the insulinase homologue Iph1 in yeast loses catalytic activity when the E^71^ active site is changed to D^71^ (36). Although we have not formally demonstrated that the INS1^m^ enzyme has lost catalytic activity, the complete replacement of the HXXEH site with AAAAA is consistent with that interpretation. Hence, the similar phenotypes of the Δ*ins1* strain and the *INS1^m^* active-site mutants suggest that the observed phenotype of the knockout is due to the loss of catalytic activity. We also observed that the defect in the *INS1^m^* active site was approximately 2 times more severe in terms of reduced oocyst shedding than the complete deletion, suggesting the expression of an inactive enzyme has a dominant-negative effect that is greater than the loss of the enzyme. Both the Δ*ins1* and the *INS1^m^* active-site mutants were less virulent in Ifngr1^−/−^ and GKO mice, and reduced oocyst shedding was associated with survival beyond 12 days, when mice infected with epitope-tagged or GFP-tagged parasites succumbed to infection. The lower virulence of the INS1 mutants is likely an indirect consequence of reduced macrogamonts, fewer oocysts, and possibly fewer thin-walled oocysts that are thought to result in reinfection, since pathology caused by the infection is normally associated with multiple rounds of merogony.

Our studies reveal that INS1 participates in macrogamont formation or maturation and that the catalytic function of INS1 is necessary for the optimal formation of oocysts. *Cryptosporidium* oocysts are comprised of an inner layer of glycoproteins, called oocyst wall proteins (OWPs), that are rich in cysteine and histidine (37) in addition to an outer acid-fast lipid layer (38). The process by which these components are synthesized, exported, and assembled into layers in the wall is largely unknown. In addition to providing insight into the development of macrogamonts, further study of INS1, and related peptidases, may reveal further insights into the process of oocyst wall formation.

## MATERIALS AND METHODS

### Animal studies.

Animal studies on mice were approved by the Institutional Animal Studies Committee (School of Medicine, Washington University in St. Louis). The Ifngr1^−/−^ mice (003288; Jackson Laboratories), Ifng^−/−^ mice (referred to as GKO) (002287; Jackson Laboratories), and *Nod scid gamma* mice (referred to as NSG) (005557; Jackson Laboratories) were bred in-house at Washington University School of Medicine and were separated by sex after weaning. Ifngr1^−/−^ and GKO mice raised in our facility are highly susceptible to the strain of C. parvum used here, and they shed high numbers of oocysts and routinely die within 10 to 12 dpi. As such, they are used to amplify transgenic strains following initial transfection of sporozoites. In contrast, NSG mice are more resistant and can be used for prolonged oocyst shedding, which occurs are lower peak levels. Mice were reared in a specific-pathogen-free facility on a 12-h:12-h light-dark cycle and water *ad libitum*. For selection and amplification of transgenic C. parvum parasites, 8- to 12-week-old mice were used and water was replaced with filtered tap water containing 16 g/liter paromomycin sulfate salt (Sigma). During the course of infection, animals that lost more than 20% of their body weight or became nonambulatory were humanely euthanized.

### Phylogenetic analysis.

The amino acid sequences of INS in Cryptosporidium parvum were extracted from CryptoDB (https://cryptodb.org/cryptodb/app), and the human insulinase was extracted from UniProt (https://www.uniprot.org). The phylogenetic tree was constructed based on these sequences. MUSCLE was used to align the concatenated sequences. Phylogenetic trees based on maximum likelihood were constructed with 1,000 replications for bootstrapping.

### Primers.

All primers were synthesized by Integrated DNA Technologies and are listed in Table S1 in the supplemental material.

### Oocyst preparation and excystation.

Cryptosporidium parvum oocysts were obtained from the Witola laboratory (University of Illinois at Urbana-Champaign). The C. parvum isolate (AUCP-1) was maintained by repeated passage in male Holstein calves and purified from fecal material, as described previously (39). Purified oocysts were stored at 4°C in 50 mM Tris-10 mM EDTA (pH 7.2) for up to 6 months before use. Before infection, 1 × 10^8^ purified oocysts were diluted into 1 ml of Dulbecco’s phosphate-buffered saline (DPBS; Corning Cellgro) and treated with 3 ml of 40% bleach (containing 8.25% sodium hypochlorite) for 10 min on ice. Oocysts were then washed 4 times in DPBS containing 1% (wt/vol) bovine serum albumin (BSA; Sigma) and resuspended in 1 ml DPBS with 1% BSA. For some experiments, oocysts were excysted prior to infection by incubating the oocysts with 0.75% (wt/vol) sodium taurocholate (Sigma) at 37°C for 60 min.

### HCT-8 cell culture.

Human ileocecal adenocarcinoma cells (HCT-8 cells; ATCC CCL-244) were cultured in RPMI 1640 medium (ATCC modification; Gibco) supplemented with 10% fetal bovine serum. The HCT-8 cells were determined to be mycoplasma negative using the e-Myco plus kit (Intron Biotechnology).

### Gene expression analysis.

HCT-8 cells were grown on 6-well culture plates and incubated 24 h before infection. Monolayers were infected with excysted sporozoites and washed twice with DPBS at 2 hpi, and fresh HCT-8 medium then was added. RNA was collected from three wells per time point in RLT buffer (Qiagen) plus 1% β-mercaptoethanol, homogenized using a QIAshredder column (Qiagen), and then stored at −80°C until further processing. RNA was extracted using the RNeasy minikit (Qiagen), treated with the DNA-free DNA removal kit (Thermo Fisher Scientific), and converted to cDNA using the SuperScript VILO cDNA synthesis kit (Thermo Fisher Scientific). Reverse transcription quantitative PCR (RT-qPCR) was performed using a QuantStudio 3 real-time PCR system (Thermo Fisher Scientific) with SYBR green JumpStart *Taq* ReadyMix (Sigma) using primers listed in Table S1. The following conditions were used for RT-qPCR: priming at 95°C for 2 min, followed by 40 cycles of denaturing at 95°C for 10 s, annealing at 60°C for 20 s, and extension at 72°C for 30 s, followed by a melt curve analysis to detect nonspecific amplification. Relative gene expression was calculated with the ΔΔ*C_T_* method (40) using C. parvum 18S rRNA as the reference gene.

### Gene tagging using CRISPR/Cas9.

To provide a single guide RNA (sgRNA) plasmid for INS1, the plasmid pACT1:Cas9-GFP, U6:sgINS1 was generated by replacing the TK sgRNA in pACT1:Cas9-GFP, U6:sgTK plasmid (12) with an sgRNA matching a region 158 bp before the stop codon in the INS1 gene (*cgd1_1680*), using Q5 site-directed mutagenesis (New England Biosciences). The INS1 sgRNA was designed using the eukaryotic pathogen CRISPR guide RNA/DNA design tool (http://grna.ctegd.uga.edu). To generate a tagging plasmid, a portion of the INS1 C terminus (253 bp) with the mutant protospacer adjacent motif (PAM) and INS1 3′UTR (117 bp) was amplified from C. parvum genome DNA. The triple hemagglutinin (3HA) epitope tag was amplified from pTUB1:YFP-mAID-3HA, DHFR-TS:HXGPRT (41). The previously described Nluc-P2A-neo^R^ reporter including the pUC19 backbone was amplified from TK-GFP-Nluc-P2A-neo-TK plasmid (12). The tagging plasmid pINS1-3HA-Nluc-P2A-neo was then generated by Gibson assembly of the components listed above. To generate a C-terminal tag with a green fluorescent protein (GFP) tag, the plasmid pINS1-GFP-Nluc-P2A-neo was constructed by swapping the 3HA with GFP from TK-GFP-Nluc-P2A-neo-TK plasmid using Gibson assembly of PCR-amplified fragments.

### Gene deletion using CRISPR/Cas9.

To provide a plasmid for gene deletion, a second sgRNA, U6:sgINS1(2), was inserted in the plasmid pACT1:Cas9-GFP, U6:sgINS1 to generate the plasmid pACT1:Cas9-GFP, dual, U6:sgINS1-KO. This plasmid contains two sgRNAs that flank the gene, one located at the C terminus of INS1, 158 bp before the stop codon, and one located at the N terminus of INS1, 55 bp after the promoter. The targeting plasmid pINS1-mCh-Nluc-P2A-neo-INS1 was made by replacing the UPRT homologous flanks with INS1 homologous flanks (770 bp upstream and 852 bp downstream of *cgd1_1680*) from UPRT-mCh-Nluc-P2A-neo-UPRT (12) using Gibson assembly of PCR-amplified fragments.

### Generating point mutations using CRISPR/Cas9.

To generate an active-site mutant of INS1, the plasmid pACT1:Cas9-GFP, dual, U6:sgINS1-mu was generated by inserting two new sgRNAs in the pACT1:Cas9-GFP, U6:sgTK plasmid. This plasmid contains two sgRNAs that flank the gene, one located at the N terminus of INS1, 5 bp after the active site, and one is located at the 3′UTR of INS1, 3 bp after the stop codon. To generate the targeting plasmid, a portion of the C terminus INS1 homologous flanks with flanking regions before the active site (3,081 bp of *cgd1_1680*) and 117-bp INS1 3′UTR was amplified from C. parvum genome DNA, and PAM sequences were mutated to prevent recutting the repair DNA. The active site in INS1 HLIEH was then mutated to AAAAA using Q5 site-directed mutagenesis. The 3HA-Nluc-P2A-neo^R^ reporter including the pUC19 backbone was amplified from INS1-3HA-Nluc-P2A-neo plasmid. The targeting plasmid pINS1(mu)-3HA-Nluc-P2A-neo was generated by Gibson assembly of the components listed above.

### Transfection of C. parvum sporozoites.

Oocysts (1.25 × 10^7^ per transfection) were excysted as described above, and sporozoites were pelleted by centrifugation and resuspended in SF buffer (Lonza) containing 50 μg of tagging or targeting plasmids and 30 μg CRISPR/Cas9 plasmid in a total volume of 100 μl. The mixtures were then transferred to a 100-μl cuvette (Lonza) and electroporated on an AMAXA 4D-Nucleofector system (Lonza) using program EH100. Electroporated sporozoites were transferred to cold DPBS and kept on ice before infecting mice.

### Selection and amplification of transgenic parasites in immunodeficient mice.

Three Infgr1^−/−^ mice were used for the first round of transgenic parasite selection. Each mouse was orally gavaged with 200 μl of saturated sodium bicarbonate 5 min prior to infection. Each mouse was then gavaged with 2.5 × 10^7^ electroporated sporozoites. All mice received drinking water with 16 g/liter paromomycin continuously from the first day postinfection (dpi), based on previously published protocols (13). Fecal pellets were collected begin at 9 to 15 dpi, after which animals were euthanized by CO_2_ asphyxiation according to the animal protocol guidelines. Fecal pellets were stored at −80°C for qPCR or at 4°C for luciferase assays or for isolating oocysts for subsequent infections.

A second round of amplification was performed by orally gavaging 3 to 4 NSG mice (used for isolating tagged strains) or GKO mice (used for amplifying knockout or mutation lines) using a fecal slurry from round one mice described above. The fecal pellets were transferred to a 1.7-ml microcentrifuge tube, ground with a pestle, diluted by addition of 1 ml cold DPBS, vortexed for 30 s, and centrifuging at 200 rpm for 10 min to pellet large particulates. The supernatant was then diluted in DPBS to achieve a concentration of ∼2 × 10^4^ oocysts in 200 μl DPBS and then gavaged into one mouse. Similar to round one, the mice infected in round two were treated with 16 g/liter paromomycin drinking water for the entirety of the experiment. Fecal pellets for qPCR and luciferase assay were collected every 3 days starting 3 dpi and fecal pellets for purification were collected every day starting at 12 dpi and stored at 4°C. For purification, fecal samples from all mice were pooled and oocysts extracted as previously described (42). Purified oocysts were stored in PBS at 4°C and used within 6 months of extraction.

### Luciferase assay.

Luciferase assays were performed with the Nano-Glo Luciferase assay kit (Promega). Mouse fecal pellets were collected in 1.7-ml microcentrifuge tubes, ground with a pestle, and then 3-mm glass beads (Fisher Scientific) and 1 ml fecal lysis buffer (50 mM Tris pH 7.6, 2 mM DTT, 2 mM EDTA pH 8.0, 10% glycerol, 1% Triton X-100 prepared in water) (43) were added to the tube. Tubes were incubated at 4°C for 30 min, vortexed for 1 min, and then spun at 16,000 × *g* for 1 min to pellet debris. The 100-μl supernatant was added split between two wells of a 96-well white plate (Costar 3610), and then 100 μl of a 25:1 Nano-Glo Luciferase buffer to Nano-Glo Luciferase substrate mix was added to each well, and the plate was incubated for 3 min at room temperature. Luminescence values were read on a Cytation 3 cell imaging multi-mode reader (BioTek).

### Fecal DNA extraction and quantification of oocysts.

DNA was extracted from fecal pellets using the QIAamp PowerFecal DNA kit (Qiagen) according to the manufacturer’s protocols. Oocyst numbers were quantified using qPCR with the C. parvum glyceraldehyde-3-phosphate dehydrogenase (GAPDH) primers (Table S1), as described previously (12). A standard curve for C. parvum genomic DNA was generated by purifying DNA from a known number of oocysts and creating a dilution series. Reactions were performed on a QuantStudio 3 real-time PCR system (Thermo Fisher) with the amplification conditions as previously described (12).

### PCR identification of transgenic parasites.

To check for the successful insertion of the target sequence into the *INS1* locus, PCR was performed on 1 μl purified fecal DNA using Q5 Hot Start high-fidelity 2× master mix (New England Biosciences) with primers listed in Table S1 at a final concentration of 500 nM each. PCRs were performed on a Veriti 96-well thermal cycler (Applied Biosystems) with the following cycling conditions: 98°C for 30 s, followed by 35 cycles of 98°C for 15 s, 60°C for 30 s, and 72°C for 2 min, with a final extension of 72°C for 2 min. PCR products were resolved on 1.0% agarose gel containing GelRed (diluted 1:10,000; Biotium) and imaged on a ChemiDoc MP imaging system (Bio-Rad).

### Indirect immunofluorescence microscopy.

HCT-8 cells grown on coverslips were infected 24 h postseeding with 1 × 10^5^ oocysts per well and then fixed with 4% formaldehyde at specific time points. The fixed samples were washed twice with DPBS and then permeabilized and blocked with DPBS containing 1% BSA and 0.1% Triton X-100 (Sigma). Primary antibodies were diluted in blocking buffer for staining: rat anti-HA was used at 1:500, rabbit anti-GFP was used at 1:1,000, MAb 4D8 (hybridoma supernatant) was used at 1:20, MAb OW50 (10) was used at 1:10, and pan-Cp (rabbit or rat polyclonal antibody) was used at 1:10,000. Cells were incubated with primary antibodies for 60 min at room temperature, washed three times with PBS, and then incubated for 60 min at room temperature in secondary antibodies conjugated to Alexa Fluor dyes (Thermo Fisher Scientific) diluted 1:1,000 in blocking buffer. Nuclear DNA was stained with Hoechst (Thermo Fisher Scientific) diluted 1:1,000 in blocking buffer for 15 min at room temperature and then mounted with Prolong Diamond antifade mountant (Thermo Fisher Scientific). Imaging was performed on a Zeiss Axioskop Mot Plus fluorescence microscope equipped with a 100×, 1.4-numeric-aperture Zeiss Plan Apochromat oil objective lens and an AxioCam MRm monochrome digital camera. Images were acquired using AxioVision software (Carl Zeiss Inc.) and manipulated in ImageJ or Photoshop.

### Transmission electron microscopy.

HCT-8 cells were infected with INS1-GFP parasites for 48 h, and infected cells then were fixed in freshly prepared mixture of 4% paraformaldehyde and 0.05% glutaraldehyde (Polysciences Inc., Warrington, PA) in 100 mM piperazine-*N*,*N*′-bis(2-ethanesulfonic acid) (PIPES)–0.5 mM MgCl_2_ buffer (pH 7.2) for 60 min at 4°C. Samples were then embedded in 10% gelatin and infiltrated overnight with 2.3 M sucrose–20% polyvinyl pyrrolidone in PIPES–MgCl_2_ at 4°C. Samples were trimmed, frozen in liquid nitrogen, and sectioned with a Leica Ultracut UCT7 cryo-ultramicrotome (Leica Microsystems Inc., Bannockburn, IL). Ultrathin sections of 50 nm were blocked with 5% fetal bovine serum–5% normal goat serum for 30 min and subsequently incubated with rabbit anti-GFP antibody (Life Technologies Corp., Eugene, OR) for 60 min at room temperature. Following washes in block buffer, sections were incubated with goat anti-rabbit IgG (H+L) conjugated to 18-nm colloidal gold (Jackson ImmunoResearch Laboratories, Inc., West Grove, PA) for 60 min. Sections were stained with 0.3% uranyl acetate–2% methyl cellulose and viewed on a JEOL 1200 EX transmission electron microscope (JEOL USA Inc., Peabody, MA) equipped with an AMT 8 megapixel digital camera and AMT Image Capture Engine V602 software (Advanced Microscopy Techniques, Woburn, MA). All labeling experiments were conducted in parallel with controls omitting the primary antibody. These controls were consistently negative at the concentration of colloidal gold conjugated secondary antibodies used in these studies.

### Quantification and statistical analysis.

All statistical analyses were performed in GraphPad Prism 8 (GraphPad Software) unless otherwise specified. When comparing the means of two groups at the same time point, we used an unpaired *t* test. For statistical analysis, *P* values of ≤0.05 were considered significant.

### Data availability.

All of the data are found in the manuscript or supplemental material.
