# Supplementary material for: Insulinase-like Protease 1 Contributes to Macrogamont Formation in Cryptosporidium parvum
Source: mBio. 2021 Mar 9;12(2):e03405-20. doi: 10.1128/mBio.03405-20 (PMC8092296; doi:10.1128/mBio.03405-20)
Supplement: FIG S1 [file mBio.03405-20-sf001.docx]

Figure S1. Selection of transgenic parasites in Ifngr1^-/-^ mice


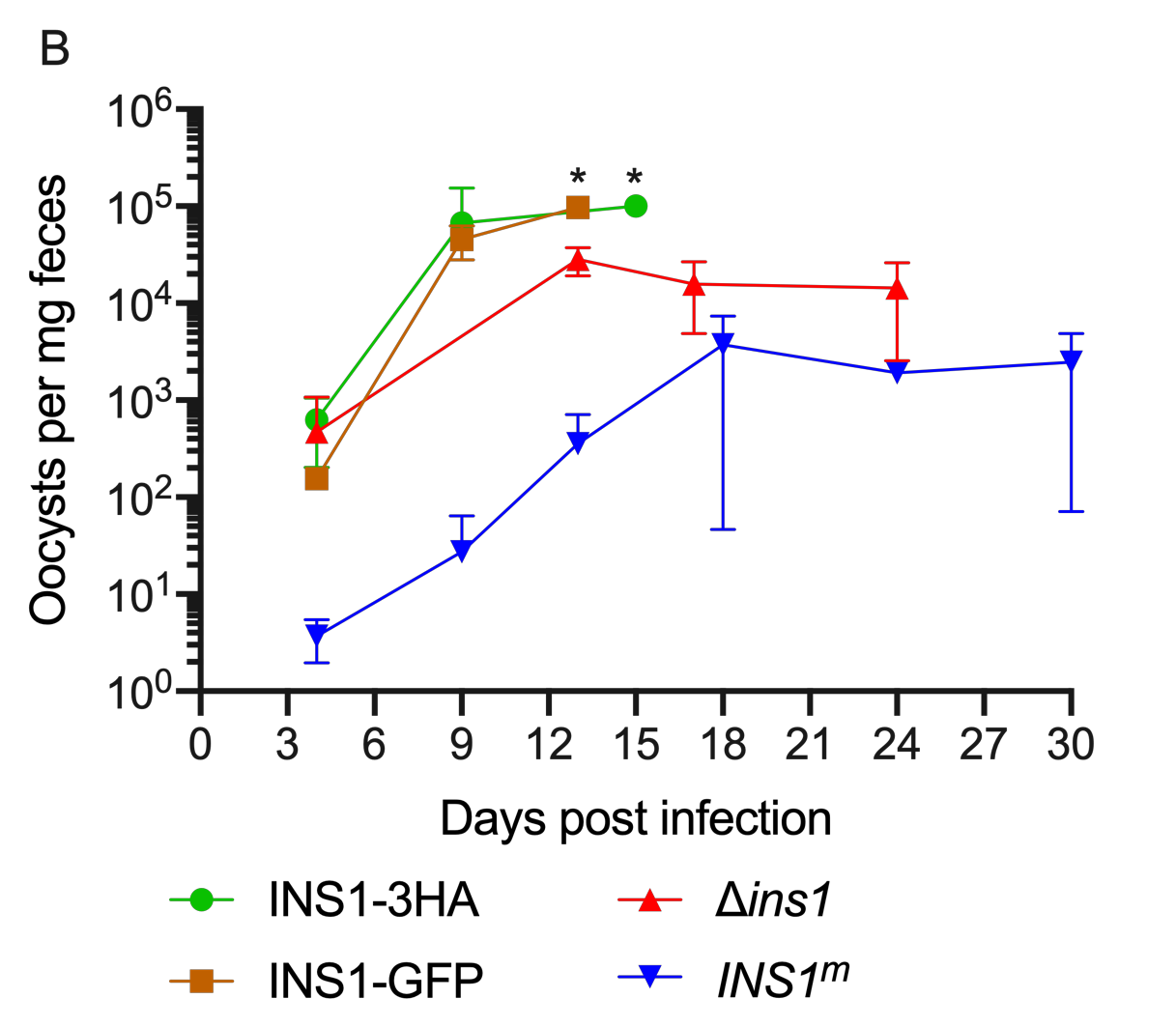

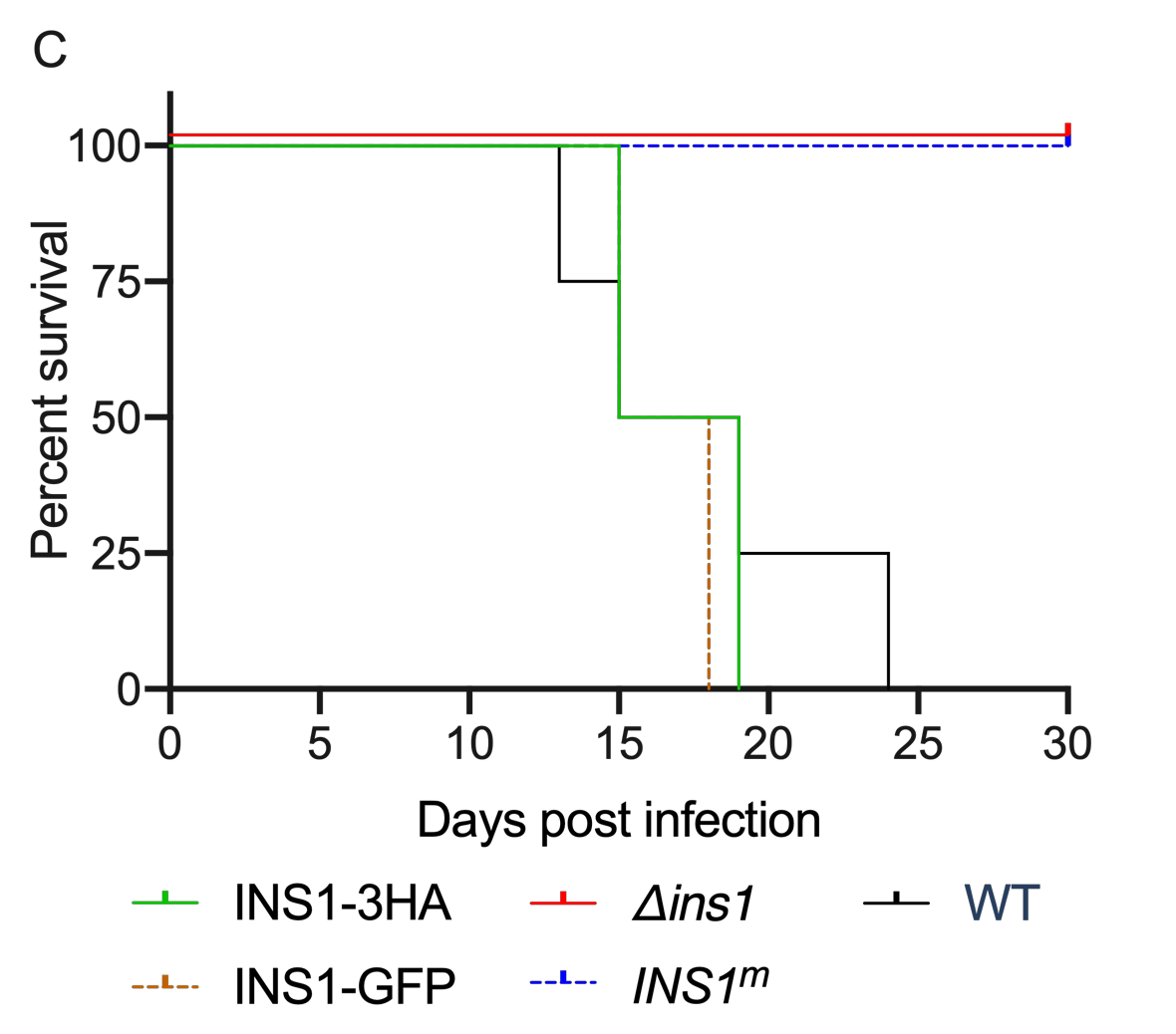
(A) Detection of NanoLuc expression from transgenic *C. parvum* oocysts in mouse fecal pellets.
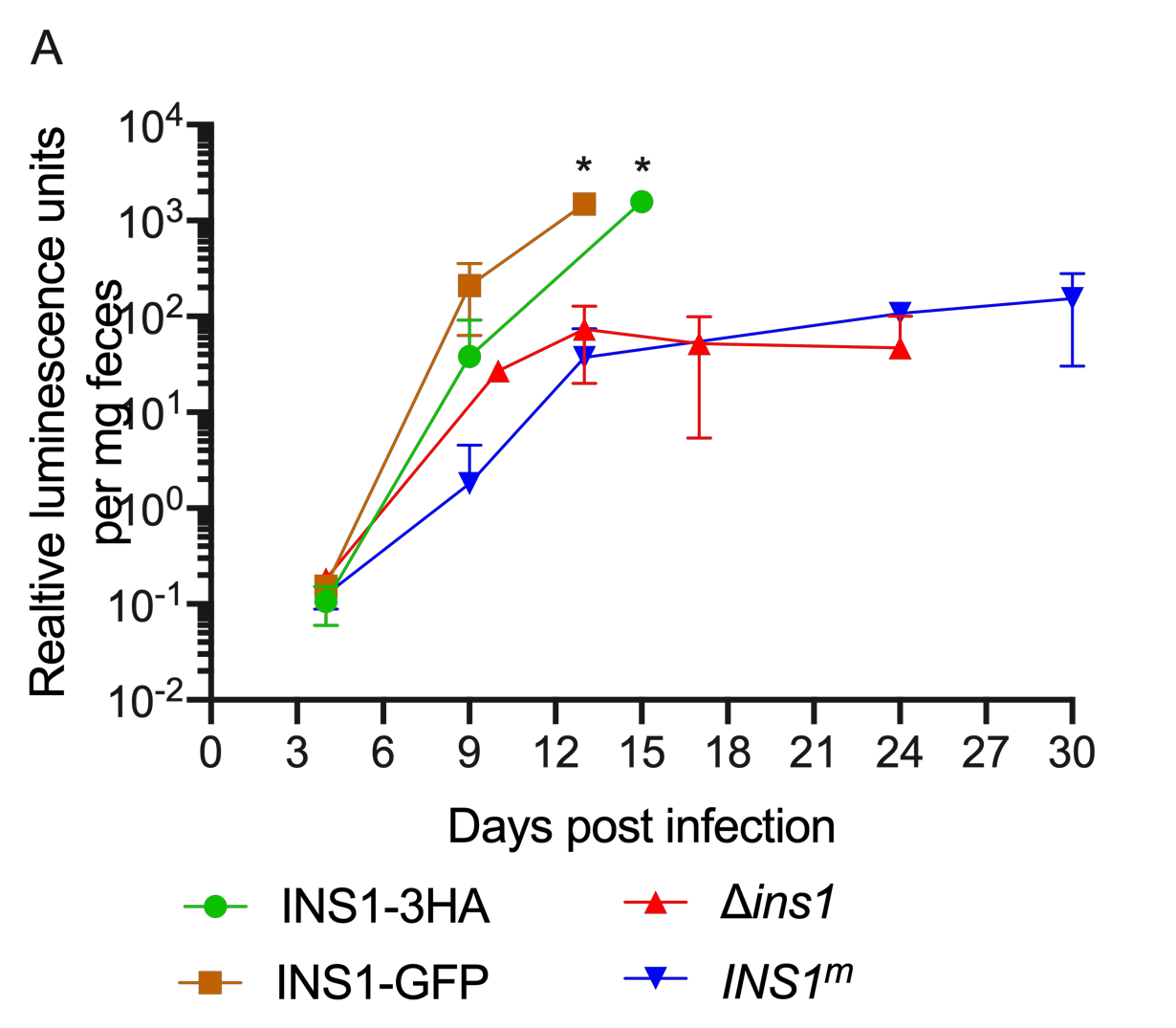
Ifngr1^-/^- mice were gavaged with 2.5 × 10^7^ transfected sporozoites of the indicated lines and mice received 16 g/L paromomycin drinking water from the first day post-infection (dpi) for the duration of the experiment. For the INS1-3HA and INS1-GFP groups, the data plotted here are the average and variance from two animals. For the ∆*ins1* and *INS1^m^* groups, the data shown are the averages and S.D. *, indicates animals that died.

(B) The number of oocysts per mg of feces was measured by qPCR. Animals in each group are the same as those shown in A. For the INS1-3HA and INS1-GFP groups, the data plotted here are the average and variance from two animals. For the ∆*ins1* and *INS1^m^* groups, the data shown are the averages and S.D. *, indicates animals that died.

(C) Survival curve of Ifngr1^-^/^-^ mice infected with different transgenic parasites. Animals correspond to those shown above in A, B. For the WT group, four Ifngr1^-/-^ mice were gavaged with 2 × 10^4^ oocysts.
